# Supplementary material for: Modeling glioblastoma heterogeneity as a dynamic network of cell states
Source: Mol Syst Biol. 2021 Sep 16;17(9):e10105. doi: 10.15252/msb.202010105 (PMC8444284; doi:10.15252/msb.202010105)
Supplement: Supplementary file 6 — Source Data for Figure 5 [file MSB-17-e10105-s004.zip › Figure5A_sourcedata/GSEA_3017/hallmarks_stateE.GseaPreranked.1621934677158/gsea_report_for_na_neg_1621934677158.html]

Report for na\_neg 1621934677158 [GSEA]

| GS  follow link to MSigDB | GS DETAILS | SIZE | ES | NES | NOM p-val | FDR q-val | FWER p-val | RANK AT MAX | LEADING EDGE || 1 | HALLMARK\_G2M\_CHECKPOINT | Details ... | 99 | -0.61 | -3.19 | 0.000 | 0.000 | 0.000 | 178 | tags=57%, list=19%, signal=62% |
| 2 | HALLMARK\_E2F\_TARGETS | Details ... | 124 | -0.57 | -3.10 | 0.000 | 0.000 | 0.000 | 220 | tags=57%, list=23%, signal=65% |
| 3 | HALLMARK\_MITOTIC\_SPINDLE | Details ... | 57 | -0.58 | -2.80 | 0.000 | 0.000 | 0.000 | 159 | tags=54%, list=17%, signal=61% |
| 4 | HALLMARK\_SPERMATOGENESIS | Details ... | 17 | -0.61 | -2.12 | 0.000 | 0.001 | 0.005 | 224 | tags=71%, list=24%, signal=91% |
| 5 | HALLMARK\_MYC\_TARGETS\_V1 | Details ... | 33 | -0.46 | -1.97 | 0.001 | 0.004 | 0.029 | 175 | tags=42%, list=18%, signal=50% |
| 6 | HALLMARK\_DNA\_REPAIR | Details ... | 28 | -0.38 | -1.53 | 0.054 | 0.068 | 0.475 | 269 | tags=50%, list=28%, signal=68% |
| 7 | HALLMARK\_ESTROGEN\_RESPONSE\_LATE | Details ... | 22 | -0.24 | -0.94 | 0.532 | 0.632 | 1.000 | 9 | tags=9%, list=1%, signal=9% |
| 8 | HALLMARK\_GLYCOLYSIS | Details ... | 26 | -0.19 | -0.76 | 0.759 | 0.771 | 1.000 | 170 | tags=23%, list=18%, signal=27% |
Table: Gene sets enriched in phenotype **na **[plain text format]****
